# Selective fusion in adolescent idiopathic scoliosis: a radiographic evaluation of risk factors for imbalance

**DOI:** 10.1007/s11832-015-0653-0

**Published:** 2015-04-07

**Authors:** D. Studer, A. Awais, N. Williams, G. Antoniou, N. Eardley-Harris, P. Cundy

**Affiliations:** Orthopaedic Department, University Children’s Hospital, PO Box 4031, Basel, Switzerland; Orthopaedic Department, Women’s and Children’s Hospital, Adelaide, Australia

**Keywords:** Selective spinal fusion, Adolescent idiopathic scoliosis, Complications

## Abstract

**Study design:**

Retrospective database, chart and medical imaging review.

**Objectives:**

To report on the outcome and evaluate possible risk factors for postoperative complications following selective spinal fusion in patients with adolescent idiopathic scoliosis (AIS).

**Materials and methods:**

All patients with AIS who underwent either a selective thoracic or selective thoracolumbar/lumbar spinal fusion at our institution from January 2001 to December 2011 inclusive were included in this study. The minimum postoperative follow-up period of all patients was 2 years.

**Results:**

During the 11-year study period, 157 patients with AIS underwent surgery for their progressive spinal deformity. Thirty patients (19 %) had a selective spinal fusion, with 16 patients (group A) having a selective thoracic, and 14 patients (group B) having a selective thoracolumbar/lumbar spinal arthrodesis. In both groups the main postoperative complications were adding-on (25 % group A, 36 % group B) and coronal decompensation (25 % group A, 29 % group B). In group A, no statistically significant risk factors for postoperative complications were identified. In group B, global coronal balance was identified as a significant risk factor for adding-on. Patients with adding-on had significantly higher coronal balance scores (mean 3.6) than those who did not experience adding-on (mean 1.9) (*p* = 0.03). In addition, those with adding-on had a significantly smaller bending lumbar Cobb angle (mean 15) than those without adding-on (mean 31.6) (*p* = 0.015). None of the patients who underwent selective spinal fusion required revision surgery.

**Conclusion:**

Although the complication rate after performing a selective spinal fusion is high, the revision rate remains low and the debate whether or not to perform a selective spinal fusion will continue.

## Introduction

Although a diagnosis of exclusion, idiopathic scoliosis (IS) is responsible for approximately 80 % of all coronal plane spinal deformities [1]. The main goal in the surgical treatment of IS should be to optimize coronal and sagittal balance and avoid further curve progression. Ideally, this can be achieved by correcting the deformity while fusing the lowest number of mobile segments and avoiding any complications, such as junctional kyphosis, adding-on or revision surgery [2–5].

There has been considerable debate regarding the appropriateness of selective spinal fusion for adolescent idiopathic scoliosis (AIS). The point of contention is whether a more rigid and straighter spine or a mobile and less straight spine provides better outcomes [2].

In 1983 King et al. [6] were the first to recommend specific vertebral levels to be included in a spinal arthrodesis, based on their classification system for AIS. These recommendations guided spine surgeons in their surgical treatment of AIS for almost two decades. Changes in operative techniques, increasing criticism with respect to inter-observer and intra-observer reliability and the fact that it essentially focused on thoracic curves led to a need for the development of a more generic classification for all curve types [7–9]. In 2001, Lenke and colleagues presented a new classification system for AIS that has been statistically proven to have improved reliability and reproducibility and provided an analysis of not only the thoracic spine but also the thoracolumbar and lumbar curve types [10]. This classification comprises a three-tiered analysis of curves based on curve type, lumbar modifier and sagittal modifier and requires standing coronal and lateral full spine radiographs as well as supine side-bending films. It begins with an evaluation of the three major spinal column regions: proximal thoracic (PT), main thoracic (MT) and thoracolumbar/lumbar (TL/L). The major curve has the largest Cobb angle and will always be included in the fusion. Whether or not the minor curves should be fused depends on their flexibility and on how the deformity affects the sagittal plane. If a minor curve corrects to <25° on coronal side-bending films and if, in addition, the kyphosis between T2–T5 and T10–L2 is <20°, the curve is regarded as being non-structural and does not have to be included in the fusion because spontaneous coronal correction after selective fusion of the major curve is expected [6, 11]. According to the definition, a selective fusion is performed when both the thoracic and thoracolumbar/lumbar curves deviate completely from the midline, but only the major curve is fused, leaving the minor curve(s) unfused and mobile [12].

Performing a selective spinal fusion is predicted by the classification for curve types 1C (main thoracic), 2C (double thoracic) and 5C (thoracolumbar/lumbar). Curve types 3C (double major) and 6C (thoracolumbar/lumbar-main thoracic) require additional radiographic and clinical information, such as Cobb magnitude, apical vertebral translation (AVT) and apical vertebral rotation (AVR) and their respective ratio. According to Lenke et al. [12], a successful selective thoracic fusion can be achieved if the ratios of MT:TL/L Cobb angle, AVT-MT:AVT-TL/L and AVR-MT:AVR-TL/L are >1.2. Conversely, the recommended ratios for these parameters for a successful selective TL/L fusion should be >1.25. Finally, all radiological information should be confirmed by the findings of the clinical examination.

However, these treatment guidelines are not routinely accepted. Newton et al. [13] reported that only two-thirds of experienced surgeons would perform a selective thoracic fusion in Lenke 1C curves, and more recently Crawford et al. [14] documented that only 49 % (138/264) of patients with a Lenke 1C curve type underwent a selective thoracic fusion in their series. In addition, many of the articles dealing with selective spinal fusion also included curves with lumbar modifier B—sometimes even lumbar modifier A—in their analysis for selective thoracic fusion [3, 4, 15–17].

Selective fusion maintains the option to extend the fusion either to the lumbar spine after selective thoracic fusion or to the thoracic spine after selective thoracolumbar/lumbar fusion when the non-instrumented curve is found to be progressing. The main complications with selective fusion are (1) postoperative coronal decompensation (distance between C7 plumb line and CSVL >2 cm), which is found in 4–41 % of cases, (2) progression of the deformity of the unfused part of the spine, or (3) adding-on phenomenon [2, 18–20]. Adding-on is described as progression or extension of the primary curve after fusion [17].

The aim of this study was to assess the results and outcomes of surgical treatment for AIS with either a selective thoracic or a selective thoracolumbar/lumbar fusion and evaluate possible risk factors for complications after selective spinal fusion surgery.

## Materials and methods

The study setting was the Women’s and Children’s Hospital, North Adelaide, Australia, a tertiary referral paediatric hospital. Institutional Review Board approval for the study protocol was obtained. All data were retrospectively retrieved from a prospectively updated database maintained by the Department of Orthopaedic Surgery of all paediatric patients undergoing spinal correction surgery in the state of South Australia. Patients chosen for this study had a diagnosis of AIS and were divided into two groups: group (A) having undergone selective thoracic spinal fusion only and group (B) with selective thoracolumbar/lumbar spinal fusion.

**Fig. 1:**
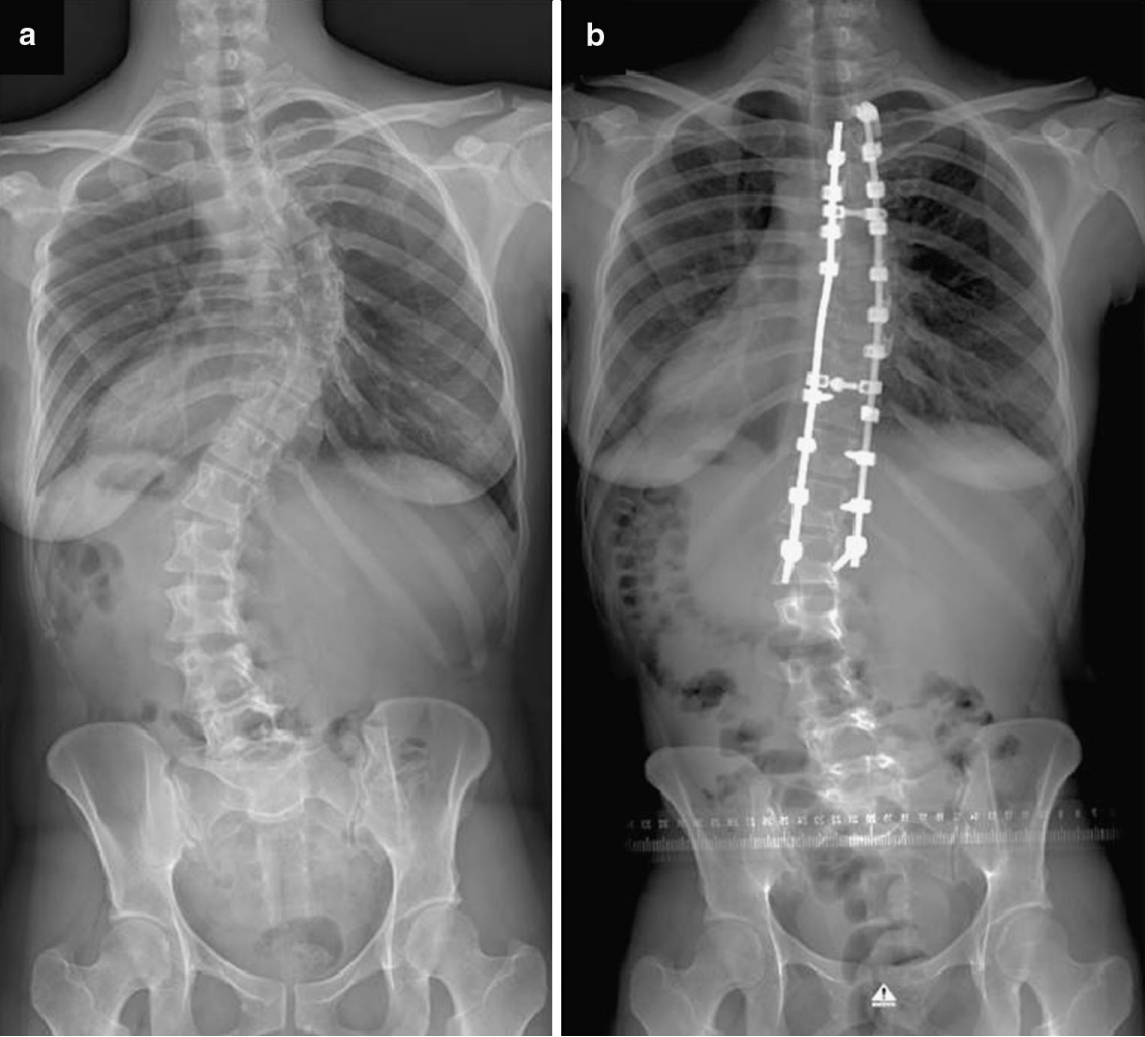
Female patient with adolescent idiopathic scoliosis Lenke type 1CN before (**a**) and after (**b**) selective thoracic posterior instrumented spinal fusion

**Fig. 2:**
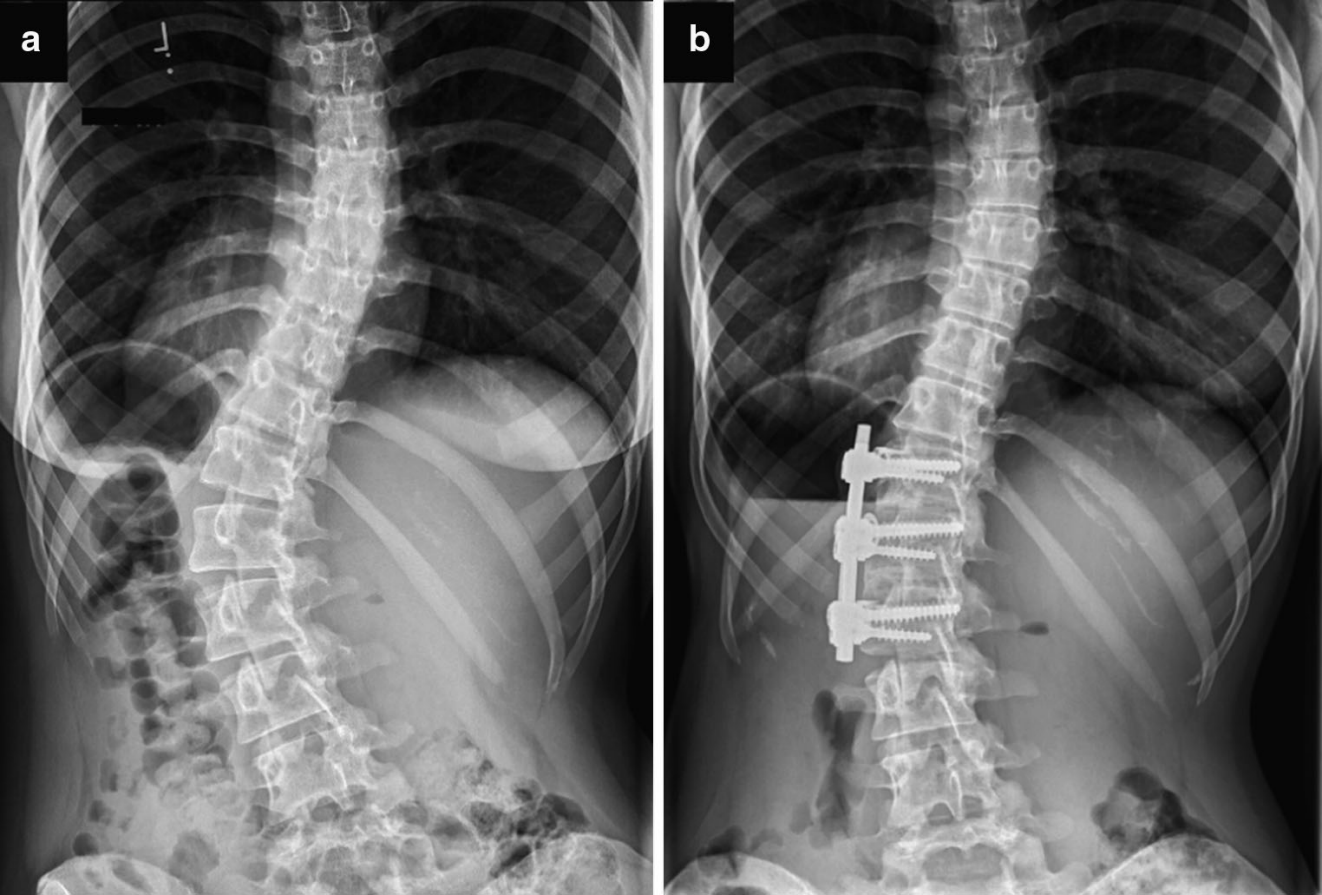
Female patient with adolescent idiopathic scoliosis Lenke type 5CN before (**a**) and after (**b**) selective thoracolumbar anterior instrumented spinal fusion

A selective thoracic fusion was defined as a fusion in which only the thoracic curve(s) was instrumented and distal fixation terminated at or above the first lumbar vertebra (L1) in patients with lumbar modifier B or C [21]. A selective thoracolumbar/lumbar fusion was defined as a fusion in which only the thoracolumbar/lumbar curve was instrumented and proximal fixation terminated at or below the ninth thoracic vertebra (T9) [21]. In group (A) patients with lumbar modifier A, and in both groups any patients with prior spinal surgery, were excluded. All selective thoracic spinal fusions were performed via a posterior approach using hybrid constructs with pedicle screws and hooks for instrumentation (Fig. 1). All patients having a selective thoracolumbar/lumbar spinal fusion underwent an anterior instrumentation with double rod screw fixation (Fig. 2).

Curve types were classified according to the Lenke classification based on preoperative standing coronal and lateral radiographs as well as supine side-bending films. All patients had a follow-up of at least 2 years with radiographs taken immediately after surgery, 1 year and 2 years postoperatively.

Apart from the Cobb angles of the three spinal regions (PT, MT, TL/L), additional documented radiographic parameters were: the Risser sign, AVT (apical vertebral translation), AVR (apical vertebral rotation), coronal and sagittal balance, thoracic kyphosis (T5–T12), thoracolumbar sagittal alignment (L10–T2), lumbar lordosis (T12–S1) and lumbo-sacral take-off angle (LSTOA), as well as the end vertebra (EV), neutral vertebra (NV) and stable vertebra (SV) of the major curve and the lowest instrumented vertebra (LIV).

The flexibility index was calculated for both groups according to King et al. [6]. AVT and AVR were measured as described by Lenke et al. [12] for the MT and the TL/L curve. Coronal balance was measured as the distance between C7 plumb line (C7PL) and the central sacral vertical line (CSVL), and sagittal balance was measured as the distance between the superior posterior corner of S1 and the C7PL. The LSTOA is the angle between the CSVL and a line through the midpoints of L4–S1 [3].

### Data analysis

Stata Intercooled v12.1 for Windows was used for all statistical analyses. Within-group comparisons were performed using the paired samples *t* test. Between-group comparisons of continuous variables were performed using one-way analysis of variance or the Wilcoxon rank-sum test if data were significantly skewed. Fisher’s exact test was used to test the association between categorical variables and Pearson’s pairwise correlation was used to examine the relationship between two continuous variables.

## Results

Between January 2001 and December 2011 inclusive, 157 patients underwent surgery for progressive AIS at the Women’s and Children’s Hospital in Adelaide, Australia. A total of 30 patients (19 %) underwent a selective spinal fusion, 16 patients (15 female, 1 male) with either lumbar modifier B or C underwent a selective thoracic spinal fusion (group A), and 14 patients (13 female, 1 male) underwent a selective thoracolumbar/lumbar arthrodesis of the spine (group B).

### Group (A): selective thoracic fusion

**Fig. 3:**
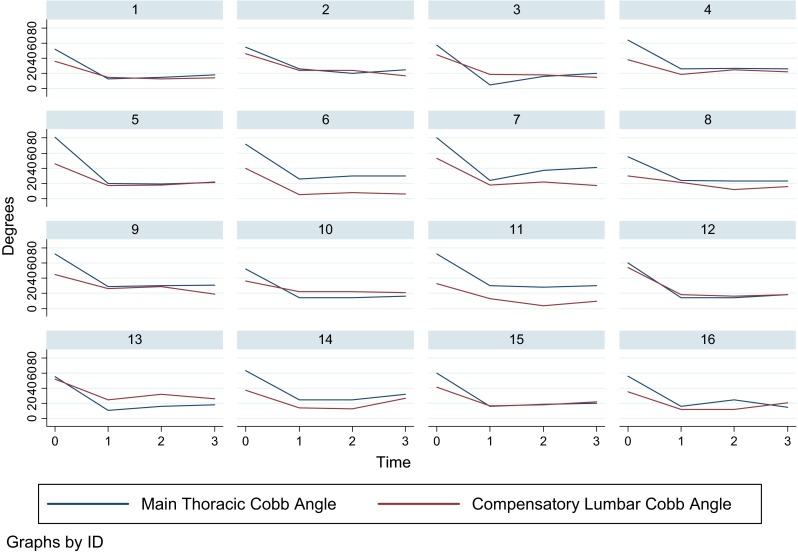
Main thoracic (*blue*) and compensatory thoracolumbar/lumbar (*red*) Cobb angle preoperatively, immediately postoperatively and 1 and 2 years after selective thoracic instrumented spinal fusion surgery

Mean age at the time of surgery was 14.7 years (range 11.3–18.5). Examination of skeletal maturity based on the Risser sign revealed that 50 % of patients had a score of 4 or 5 [0 (*n* = 1), 1 (*n* = 2), 2 (*n* = 2), 3 (*n* = 3), 4 (*n* = 7), 5 (*n* = 1)]. Curve types according to the Lenke classification were: 1B (*n* = 4), 1C (*n* = 9), 2B (*n* = 2), 3C (*n* = 1). Mean preoperative MT Cobb angle was 63° (range 52°–81°), corrected to an average of 20° immediately postoperatively, and to 24° at 2-year follow-up. Mean compensatory TL/L Cobb angle was 42° (range 30°–54°), decreasing to an average of 18° immediately postoperatively, remaining the same at the 2-year follow-up (Fig. 3).

The preoperative ratio of MT:TL/L Cobb magnitude was 1.54. Mean preoperative AVT-MT was 52 mm (range 24–92 mm) and mean AVT-TL/L was 21 mm (range 7–34 mm), with an AVT ratio of 2.91. Preoperative AVR-MT averaged 2.37, and 1.4 for AVR-TL/L, resulting in a ratio of 1.69. No patient showed a greater Cobb magnitude or AVR for the compensatory TL/L curve compared to the MT curve. Only one patient had a greater AVT of the compensatory TL/L curve.

There was no significant change in the sagittal alignment after selective thoracic spinal fusion. Mean thoracic kyphosis was 19° preoperatively and 23° at latest follow-up (*p* = 0.09); lumbar lordosis was 56° preoperatively and decreased to 50° 2 years postoperatively (*p* = 0.17). Average preoperative LSTOA was 11.4°.

Four patients (25 %) showed postoperative adding-on of their MT curve. Two of them had a 7° increase in the MT Cobb angle (11° → 18°; 25° → 32°) after selective thoracic fusion from immediately postoperatively to the 2-year follow-up. The initial Cobb angle before surgery was 55° (Lenke 3CN) and 63° (Lenke 1BN); their age at the time of surgery was 14 and 11 years, and their Risser sign 4 and 0, respectively. One patient showed an increase from 5° to 20° curve magnitude. Her initial MT Cobb angle was 57° (Lenke 1BN); her age at surgery was 15 years, and Risser sign 4. One girl had a 17° change in Cobb angle from 24° to 41°. She had a Lenke type 1CN curve with an initial Cobb angle of 80°; her age at surgery was 13 years, and Risser sign 1. Only the patient with an increase in the MT Cobb angle from 25° to 32° also showed a relevant (>5°) postoperative increase in the magnitude of the compensatory lumbar curve from 14° to 27°.

No significant differences in preoperative sagittal alignment (thoracic kyphosis *p* = 0.88, lumbar lordosis *p* = 0.45), MT Cobb magnitude (*p* = 0.84), flexibility index (*p* = 0.15), LIV (*p* = 0.42), age at surgery (*p* = 0.22), Risser sign (*p* = 0.53), or global coronal (*p* = 0.64) or sagittal balance (*p* = 0.69) could be identified between patients showing adding-on of their MT curve and those without adding-on.

Average LSTOA for the patients who had adding-on for their MT curve was 13.3° compared to a mean of 10.8° for those without adding-on (*p* = 0.32). Additionally, the preoperative LSTOA was significantly positively correlated with the preoperative compensatory thoracolumbar/lumbar curve magnitude (*r* = 0.73, *p* = 0.001) (Fig. 2). This is represented by a mean magnitude of the compensatory thoracolumbar/lumbar curve of 47° Cobb angle in the patients showing postoperative adding-on compared to an average compensatory curve magnitude of 40° for the patients without adding-on (*p* = 0.11). With that, the MT:TL/L Cobb ratio is, of course, different for patients with and without adding-on, being 1.38 versus 1.59, respectively (*p* = 0.23).

Immediately postoperatively, five patients showed coronal decompensation (range 24–34 mm), all to the left side. Three of these patients remained decompensated 2 years after surgery, one of them achieved normal coronal balance and an additional one lost her coronal balance. Two years after surgery, 4/16 (25 %) patients had a trunk shift to the left side (range 21–32 mm). Only one of the patients who had coronal decompensation after 2 years was already decompensated before surgery. Two patients who showed coronal decompensation preoperatively achieved and maintained a balanced spine postoperatively.

None of the patients who presented with coronal decompensation at the 2-year follow-up showed an adding-on phenomenon.

None of the patients undergoing selective thoracic fusion for their progressive AIS required revision surgery to include the unfused compensatory TL/L curve in the spinal arthrodesis.

### Group (B): selective thoracolumbar/lumbar fusion

**Fig. 4:**
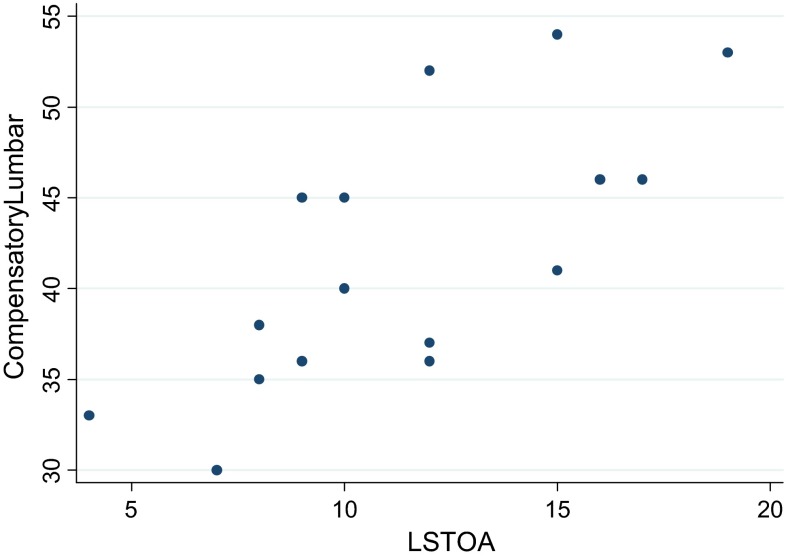
Positive correlation between lumbo-sacral take-off angle (LSTOA) and magnitude of preoperative compensatory thoracolumbar/lumbar curve in patients undergoing selective thoracic instrumented spinal fusion

Mean age at the time of surgery was 15.5 years (range 11.5–17.6). Examination of skeletal maturity revealed that almost 79 % of patients had a Risser sign of 4 or 5 [0 (*n* = 1), 1 (*n* = 1), 2 (*n* = 1), 3 (*n* = 6), 5 (*n* = 5)]. Curve types according to the Lenke system were: 5C (*n* = 13), 6C (*n* = 1). Mean preoperative TL/L Cobb angle was 54° (range 41°–78°), corrected to an average of 21° immediately postoperatively, and to 24° at 2-year follow-up. Mean compensatory thoracic Cobb angle was 28° (range 14°–52°), decreasing to an average of 19° immediately postoperatively, and slightly increasing to 24° at 2-year follow-up (Fig. 4).

The preoperative ratio of TL/L:MT Cobb magnitude was 2.16. Mean preoperative AVT-TL/L was 56 mm (range 39–68 mm) and mean AVT-MT was 16 mm (range 3–35 mm), with an AVT ratio of 6.5. Preoperative AVR-TL/L averaged 3, and 1.5 for AVR-MT, resulting in a ratio of 2.0. No patient showed a greater Cobb magnitude, AVT or AVR for the compensatory MT curve compared to the TL/L curve.

There was no significant change in the sagittal alignment after selective thoracolumbar/lumbar spinal fusion. Mean thoracic kyphosis was 24° preoperatively and 27° at latest follow-up (*p* = 0.20), lumbar lordosis was 55° preoperatively and 56° 2 years postoperatively (*p* = 0.88). Average preoperative LSTOA was 14.1°.

In this group, five patients (36 %) showed postoperative adding-on for their instrumented TL/L curve. The smallest increase was from 1° immediately postoperatively to 9° after 2 years in a girl with a Lenke 5C curve. The initial Cobb angle before surgery was 41°; her age at the time of surgery was 16 years, and Risser sign 4. For this girl the compensatory MT curve showed a relevant postoperative progression (>5°) from 2° to 12°. One girl increased her TL/L curve post-fusion from 22° to 31° (Lenke 5CN). Her unfused compensatory MT curve showed no relevant postoperative change and remained at 36°. Two girls had a postoperative increase in their TL/L curve of 11° (10° → 21°, Lenke 5CN; 21° → 32°, Lenke 5C-). In both cases the compensatory MT curve again showed a postoperative increase in magnitude from 15° to 21° and from 20° to 40°, respectively. Another girl had an increase in her TL/L curve of 13° postoperatively (22° → 35°, Lenke type 5CN). Her compensatory MT curve also increased from 6° to 16° within the 2-year follow-up period.

No significant differences in preoperative TL/L (*p* = 0.61) or compensatory MT (*p* = 0.78) Cobb magnitude, lumbar lordosis (*p* = 0.80), LSTOA (*p* = 0.94), LIV (*p* = 0.74), or sagittal balance (*p* = 0.25) could be identified between patients showing adding-on of their TL/L curve and those without adding-on. A significant difference in global coronal balance, however, was observed between those showing adding-on (mean 36 mm) and those without adding-on (mean 19 mm), (*p* = 0.026). Interestingly, comparing patients with or without adding-on, average age at surgery was 14.3 years versus 16.1 years (*p* = 0.21), average Risser sign was 2.8 versus 4.4 (*p* = 0.09), mean thoracic kyphosis was 19.6° versus 27.2° (*p* = 0.33) and both the TL/L (*p* = 0.015) and compensatory MT (*p* = 0.33) curves were more flexible on side-bending films (TL/L curves corrected to 15° versus 31.6°; compensatory MT curves corrected to 11.6° versus 16.9).

Immediately postoperatively 50 % (7/14) of the patients showed coronal decompensation (range 27–60 mm): 6/7 to the left side, and only one to the right side. At the 2-year follow-up 29 % (4/14) were decompensated (range 22–54 mm): two patients to either side (only one of these four patients was also decompensated immediately postoperatively). Two of these patients already showed coronal decompensation before surgery.

As in group (A), again none of the patients undergoing selective thoracolumbar/lumbar fusion for their progressive idiopathic scoliosis required revision surgery to include the unfused compensatory MT curve in the spinal arthrodesis.

## Discussion

Even 12 years after Lenke et al. [12] presented their new classification system for AIS there is still significant debate regarding the appropriateness of performing selective spinal fusions [13, 14, 20]. Between 2001 and 2011 only 19 % (30/157) of our cohort underwent either a selective thoracic or thoracolumbar/lumbar spinal fusion in the surgical treatment of their idiopathic scoliosis. The fear of well-known complications, such as progression of the non-instrumented curve, adding-on of the fused area of the spine, or coronal decompensation, might be a reason for the low number of selective spinal fusions. Additionally, newer instrumentation techniques allow for a very high correction rate that may straighten the major curve beyond the non-instrumented curve’s ability to compensate, therefore requiring an “under-correction” of the major curve that might be unattractive for many surgeons [14, 22]. Another reason for the low number of selective spinal fusions in our cohort might be the fact that the mean Cobb angle of the major curve in both groups (A and B) was higher than reported elsewhere in the literature [3, 14]. This might be supported by the geographical situation in South Australia, with many rural patients presenting late with a severely progressed deformity.

### Group (A)

**Fig. 5:**
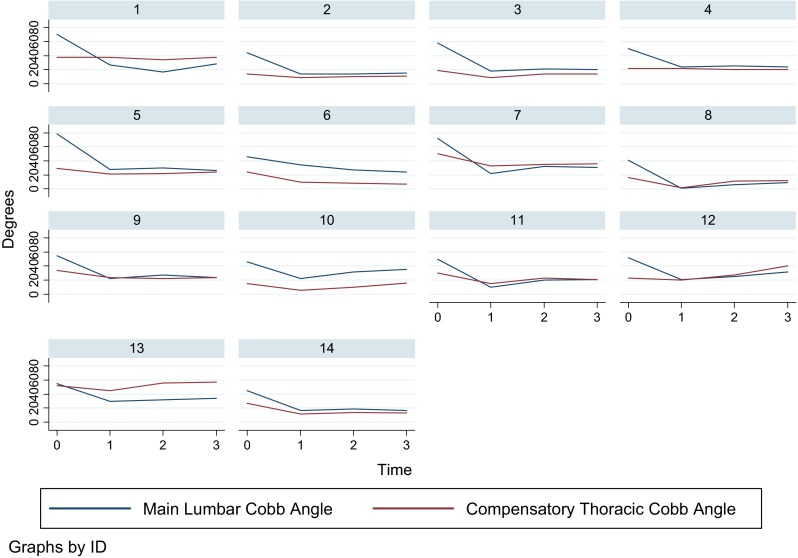
Main thoracolumbar/lumbar (*blue*) and compensatory thoracic (*red*) Cobb angle preoperatively, immediately postoperatively and 1 and 2 years after selective thoracolumbar/lumbar instrumented spinal fusion surgery

Our results are consistent with previous reports in the literature [2, 18–20], with 25 % of our patients demonstrating adding-on with a relevant progression/extension (>5°) of the instrumented major curve after performing a selective thoracic fusion. Of all recorded parameters, only the LSTOA could be identified as a possible risk factor for our patients to sustain adding-on. A greater LSTOA was significantly associated with a greater preoperative compensatory TL/L curve magnitude (Fig. 5). This, conversely, resulted in a lower average correction of the compensatory TL/L curve of 53 % in patients with adding-on, compared to a mean decrease in Cobb angle of 64 % in those patients without adding-on. Abel et al. [3] in 2011 looked at 204 patients with idiopathic scoliosis who had undergone posterior spinal arthrodesis. They compared selective versus non-selective thoracic spinal fusion and demonstrated that both groups showed a positive correlation between preoperative LSTOA and preoperative TL/L Cobb angle, and both groups significantly improved in coronal TL/L Cobb angle as well as in LSTOA postoperatively. Interestingly, in the non-selective fusion group the LSTOA decreased by an average of 11° compared to only 2° in the selective fusion group. They concluded that to appreciably change the LSTOA with a posterior spinal fusion the distal level of fixation must be beyond the apex of the TL/L curve. This again adds further controversy to the discussion of whether a stiffer straight spine leads to a better outcome than a less straight but more mobile spine.

The same rationale is reflected when looking at coronal balance. Again, 25 % of our patients showed coronal decompensation 2 years after their spinal arthrodesis. Only one of them had evidence of decompensation preoperatively. Additionally, two of the three patients who were decompensated before surgery had good coronal balance at 2-year follow-up. This is contradictory to the findings of Demura et al. [20] where 57 % of the patients with Lenke 1C curves who were decompensated after a selective thoracic fusion already showed coronal decompensation preoperatively.

### Group (B)

In this group, 36 % of our patients who underwent selective thoracolumbar/lumbar anterior spinal fusion sustained adding-on and 29 % showed coronal decompensation. Sanders et al. [23] looked at 49 patients with AIS who underwent selective thoracolumbar/lumbar instrumented spinal fusion and tried to identify factors to predict a satisfactory outcome postoperatively. A TL/L:MT Cobb ratio >1.25 in combination with a MT curve that corrects to <20° on side-bending was the best structural predictor for a good outcome. A closed triradiate cartilage was the best predictor concerning maturity. These findings are only partially reflected in our results. Patients who had postoperative adding-on in our series were also less skeletally mature, but their compensatory MT curve was more flexible, with an average correction of 59 % on side-bending compared to an average of 43 % in patients without adding-on. The influence of the preoperative sagittal alignment on the outcome after selective thoracolumbar/lumbar fusion has not been thoroughly investigated to date. Whether or not, as in our patients, a hypokyphosis of the thoracic spine can be seen as a negative predictor needs further assessment with larger patient numbers.

Given that this is a radiologic evaluation of the results after selective spinal fusion, the lack of clinical data and patient’s self-assessment/satisfaction is a limitation of this study. However, the need to proceed to revision surgery was based on clinical assessment, discussion with child and parents and the lack of progression of the adjacent curve with longer follow-up.

## Conclusion

This study provides further evidence for the success of selective spinal fusion. Although radiological evidence of adding-on and coronal imbalance was observed, this was unlikely to be clinically significant, with no cases requiring revision surgery. Factors predictive of a good outcome were normal preoperative sagittal alignment, a low LSTOA, and, as expected, being close to or having reached skeletal maturity. However, considering the small population included in our study and the consecutive lack of statistical significance, further studies with larger patient cohorts are needed to support our findings.
